# Mortality of Chicago for February, 1861

**Published:** 1861-04

**Authors:** 


### MORTALITY OF CHICAGO FOR FEBRUARY, 1861.

| DISEASE. | NO. | DISEASE. | NO. |
|---|---|---|---|
| Brain, Inflammation of... | 2 | Rheumatism............ | 2 |
| Convulsions ............ | 6 | Still Born.............. | 2 |
| Hydrocephalus ......... | 4 | Puerperal Fever.......... | 1 |
| Teething.............. | 20 | Old Age.............. | 3 |
| Exposure.............. | 1 | Burns................. | 1 |
| Diphtheria ............ | 15 | Intemperance.......... | 2 |
| Croup ................ | 9 | Typhoid Fever.......... | 4 |
| Whooping Cough....... | 1 | Lungs, Congestion of..... | 3 |
| Scarlet Fever........... | 3 | Liver, " " .... | 1 |
| Pneumonia............. | 5 | Consumption .......... | 25 |
| Cardiac Disease......... | 4 | Unknown.............. | 10 |
| Enteritis .............. | 4 | | |
| Cholera, Infantum...... | 7 | | 135 |

| AGES. | NO. |
|---|---|
| Under 1 year.......... | 31 |
| Between 1 and 5 years.. | 43 |
| " 5 " 10 " .. | 7 |
| " 10 " 20 " .. | 4 |
| " 20 " 30 " .. | 9 |
| " 30 " 40 " .. | 9 |
| " 40 " 50 " .. | 12 |
| " 50 " 60 " .. | 6 |
| " 60 " 70 " .. | 5 |
| " 70 " 80 " .. | 2 |
| Unknown ............. | 5 |
| | 135 |

*Consumption.*

| AGES. | NO. |
|---|---|
| Under 1 year........... | 2 |
| Bet. 10 and 20 years.... | 2 |
| " 20 " 30 " .... | 8 |
| " 30 " 40 " .... | 4 |
| " 40 " 50 " .... | 2 |
| " 50 " 60 " .... | 3 |
| " 60 " 70 " .... | 4 |
| | 25 |

*Teething.*

| AGES. | NO. |
|---|---|
| Under 1 year........... | 4 |
| Over 1 year............ | 16 |
| | 20 |

*Diphtheria.*

| AGES. | NO. |
|---|---|
| Under 1 year.......... | 1 |
| Betw. 1 and 5 years..... | 10 |
| " 5 " 10 " .... | 4 |
| | 15 |

*Deaths in the different Divisions of the City, divided as follows:*

| | | | |
|---|---|---|---|
| North Division.......... | 36 | West Division.......... | 53 |
| South " .......... | 46 | Total.................. | 135 |

The Mortality List of this year compares as follows with the List of Mortality for 13 years. The number of deaths for the month of February for a number of years has been as follows:

| | | | |
|---|---|---|---|
| 1847 | 32 | 1855 | 123 |
| 1848 | 41 | 1856 | 92 |
| 1849 | 36 | 1857 | 106 |
| 1850 | 53 | 1858 | 126 |
| 1851 | 38 | 1859 | 115 |
| 1852 | 42 | 1860 | 128 |
| 1853 | 81 | 1861 | 123 |
| 1854 | 95 | | |

### MEETING OF THE ALUMNI OF RUSH MEDICAL COLLEGE.

The undersigned, Alumni of Rush Medical College resident in Chicago, propose to our brethren a Re-union, to be holden in Chicago on the day immediately preceding the meeting of the National Medical Association. Believing that such a meeting would be of interest to all to whom this call is addressed, and as a similar opportunity is not likely soon to occur, we cordially invite you to participate in the pleasures of the occasion.

W. C. HUNT, WARREN MILLER,
V. L. HURLBURT, E. O. F. ROLER,
JOHN NUTT, EDWIN POWEL,
E. INGALS, I. P. LYNN,
PHILIP MATHEI, J. W. FREER,
JOHN B. BROWN.

